# “I Know They’re Going to Weaponize This:” Black and Latino Sexual Minority Men’s Mpox-Related Sexual Behaviors, Stigma Concerns, and Vaccination Barriers and Facilitators

**DOI:** 10.1007/s40615-025-02404-x

**Published:** 2025-04-04

**Authors:** Orlando O. Harris, Donte Boyd, Gamji Rabiu Abu-Ba’are, Joseph Egbunikeokye, Mitchell Wharton

**Affiliations:** 1 Department of Community Health Systems, School of Nursing, University of California, San Francisco, 490 Illinois Street, Floor 9, Box 0608, San Francisco, CA 94143-0608 USA; 2 College of Social Work, The Ohio State University, Columbus, OH USA; 3 Nursing and Public Health, University of Rochester, School of Nursing, Rochester, NY USA; 4 Us Helping Us People Into Living, Inc, Washington, DC USA

**Keywords:** Sexual minority men, Mpox, Vaccines, Stigma

## Abstract

**Background:**

The Mpox epidemic disproportionately impacted Black and Latino sexual minority men (BLSMM) in the United States, with them having the highest prevalence of disease and the lowest rates of vaccination. Despite this disparity, BLSMM perspectives on the disease, Mpox-related stigma, and inequitable rollout of and distrust in the Mpox vaccine are absent from the literature. The present study aims to describe experiences with Mpox-related sexual behaviors, stigma, and vaccine barriers and facilitators among a sample of BLSMM living in both California and New York.

**Methods:**

In this qualitative interpretive phenomenological study, we utilized semi-structured individual interviews as the primary source for data collection. Data was collected between August 2021 and December 2022 from 41 adult participants in California and New York. Interviews were recorded, transcribed verbatim, and analyzed using thematic content analysis.

**Results:**

Participants ranged in age from 19 to 65 years, with the majority identifying as Black (73%) and male gender (93%). Overall, participants’ narratives revealed that initial messaging around Mpox produced stigma parallel to the HIV/AIDS pandemic with many participants curtailing their sexual behaviors as a harm reduction strategy. Mpox-related stigma negatively impacted sexual minority communities both internally and externally. Participants’ narratives also revealed general vaccine skepticism due to existing medical distrust, negative vaccine experiences by other BLSMM, and lack of prioritization of outreach and distribution efforts in communities of color.

**Conclusion:**

Alterations to vaccine administration protocols and outreach efforts for reasons identified in this study are critical to addressing disparities in vaccine uptake among BLSMM. Public health practitioners must consider equitable frameworks, existing stigmas, and medical distrust when engaging BLSMM.

## Introduction

Mpox, a double-stranded DNA virus belonging to a family of orthopoxvirus, was declared a public health emergency of international concern by the World Health Organization in the early months of 2022 after the largest outbreak in history was observed outside of Africa l [46]. Following that announcement, in August of 2022, the United States (US) also declared Mpox as a national public health emergency. Global confirmed Mpox cases rose to over 100,000, with 220 confirmed deaths [7]. Since the start of the global outbreak, there have been 32,063 confirmed cases of Mpox and 58 deaths in the US [2, 14, 83]. The majority (95%) of the reported cases of Mpox globally and nationally occurred among sexual minority men (SMM) and through sexual contact, though many nonsexual cases have been reported [3, 4]. People living with HIV in the US made up 38% of total Mpox cases yet accounted for 94% of Mpox-related deaths [33, 47, 76]. While only accounting for a small portion of the US population, Black and Latino SMM (BLSMM) represented a disproportionate amount of the Mpox cases (57%) and 86% of the deaths [47, 75, 83], with the majority of the cases reported in California and New York [68].

Mirroring the Mpox epidemic, the HIV epidemic in the United States has also significantly impacted the lives of BLSMM. While new cases of HIV have continued to decline among SMM, largely due to access to oral and long-acting injectable biomedical prevention modalities [81], rates of HIV infection have consistently remained high among BLSMM (Centers for Disease Control, 2024a). For example, in 2022, Black and Latino SMM accounted for 74% of new HIV cases, compared to 24% among their White counterparts [17]. Additionally, BLSMM were more likely to report higher rates of HIV stigma, compared to White SMM [18]. Similarly, the novel coronavirus (SARS-CoV-2 [COVID-19]) pandemic, identified in 2020 and occurring at the same time as both the Mpox and HIV epidemic, also disproportionately impacted Black and Latino communities. Black and Latino communities accounted for a disproportionate amount of the COVID-19 cases and deaths in the United States even though they account for a small proportion of the population [11, 55]. There were several factors that have been identified as contributors to these disparities among BLSMM [44],however, longstanding structural, social, and economic factors were identified as primary contributors to these disparities [8, 28, 68, 70, 84].

The convergence of the three disease conditions (HIV, Mpox, and COVID-19) co-occurring at the same time contributed to the multiple layers of stigma affecting the sexual minority communities across the country [61]. The Mpox virus, which primarily affected the sexual networks of SMM, triggered a stigmatizing public health response at the local, state, and national levels [5, 52, 60]. Initial guidelines from the Centers for Disease Control and Prevention (CDC) suggested that SMM who are sexually active with other men, engage in multiple partnerships, diagnosed with an STI, have sex for money, have sex at commercial sex venues, or living with HIV were at highest risk of contracting Mpox [27, 76]. Additional CDC guidelines included providing all persons suspected of Mpox infection with a detailed sexual history, physical examination, and diagnostic testing for STIs [32]. However, at the healthcare system level, providers expressed concerns with the guidelines, specifically those requiring disclosure of sexual behaviors or sexual orientation, and viewed them as barriers to accessing preventative Mpox care [9, 15]. Moreover, these guidelines create a risk profile that stigmatizes sexual and gender (SGM) communities, leaving those who did not meet them to assume they were at lower risk [1, 5, 66]. In addition, many of the recommendations to counteract sexual and social contact amid the Mpox outbreak also contributed to internal and external stigma within the SGM community [26, 61]. Several studies with SMM suggested that based on public health information, men considered Mpox to be a “gay disease” and expressed concerns that the outbreak would be blamed on the community [51]. Similarly, the lesions that developed due to the infection contributed to internal SGM Mpox-related sexual stigma, suggesting an urgent exploration of public health recommendations, sexual behaviors, and SMM vaccine intentions.

Vaccinating all persons at risk for Mpox was seen as an emergency response for all public health departments nationwide [1, 13, 62]. However, several challenges materialized that hindered successful vaccination efforts, many of which directly resulted from inconsistent messaging from national and local health departments [16]. For example, the vaccination criteria, which followed the epidemiological risk profile established by the CDC, discouraged SMM who perceived themselves to not be at risk of contracting Mpox from accessing the vaccine [26, 37]. Individuals who did not have multiple sexual partners or did have sex at commercial venues might not perceive themselves to be at risk,thus, they might forgo accepting and accessing the vaccine [75]. There are few studies that have identified the correlation between sexual behaviors, substance use, and multiple partnerships as motivations to seek vaccination [41, 47]. This highlights the need for positive communication and harm reduction messages, instead of abstinence-only prevention, as a more effective strategy to curtail future infectious outbreaks. The previous approach, the implementation of strict risk profiles, only stigmatized the community [48, 76]. Another factor that may have hindered Mpox vaccine uptake is distrust in the healthcare system [22, 39, 67]. There is considerable evidence in the literature that suggests that distrust in the healthcare system is a significant barrier to vaccine acceptance among Black and Latino communities, which is inclusive of Black and Latino SMM (BLSMM) [39, 64, 67, 75].

The re-emergence of the Mpox virus and its associated impact on the lives of BLSMM requires a renewed focus on the sociostructural and environmental factors that drive sexual behaviors, stigma and discrimination, and vaccine hesitancy among this population [14, 30]. The available literature documenting many of these factors has primarily focused on White SMM, with very scant inclusion of BLSMM. Few studies have described these experiences for BLSMM [2, 30, 62]. This underscores the need for additional studies that explore the impact of the Mpox epidemic on the lives of racially and sexually marginalized men [2]. To that end, the purpose of this proposed qualitative interpretive phenomenological study was to describe BLSMM experiences with Mpox-related sexual behaviors, stigma, and vaccine barriers and facilitators. Our study was driven by two primary research questions. Broadly, what are the Mpox-related sexual behaviors, harm reduction strategies, and vaccination experiences among BLSMM? Specifically, what barriers and facilitators influence Mpox vaccine uptake in this population?

## Methods

The research was guided by an interpretive phenomenological design. We chose interpretive phenomenology as an appropriate methodology to describe the experience of the Mpox phenomenon from the perspectives of BLSMM living in California and New York. Interpretive phenomenological qualitative studies seek to shed light on the lived experiences of a subset of people within a population to highlight their experiences of a particular phenomenon through rich descriptive narratives [53, 63, 77]. Due to the newness of the Mpox epidemic of 2022 and the dearth of literature that describes Black and Latino men’s experiences with Mpox and the challenges they encountered accessing Mpox vaccines, this methodological approach provided descriptive details about their experiences from their perspectives [24, 73, 74]. Study oversight and approval were sought from the institutional review boards of both the University of California—San Francisco, in San Francisco, California (IRB #21–34,350, Reference #318,589), and the University of Rochester, in Rochester, New York (Study ID: STUDY00007264). Verbal and written informed consent were obtained from each participant before their enrollment in the study.

The study was guided by the social-ecological model (SEM) and intersectionality framework. The SEM, an adaptation of the Ecological model Bronfenbrenner introduced in the 1970s, stipulated five forces of influence on health behavior that go beyond individual health behavior (McLeroy et al., 1988a). These forces of influence include individual, interpersonal, community, institutional, and policy [34, 58]. We also utilized intersectionality as a framework to better understand how BLSMM’s Mpox experiences were shaped by their social and economic positions. The intersectional framework, which is rooted in Black feminist pedagogy and praxis [10, 21, 23], allowed us to explore how racism, homophobia, and other drivers of discrimination served as barriers to vaccination against Mpox. Applied together, both SEM and intersectionality complemented the use of interpretive phenomenology so much that it allowed the research team to understand experiences of marginalization, oppression, and discrimination [11, 21, 79].

Our study interview guide was created and structured to include each component of the SEM framework with a focus on intersectionality. First, at the individual level, we assessed BLSMM’s Mpox social and sexual behaviors and internalized manifestations of Mpox-related stigma. At the interpersonal level, we explored participants’ social and sexual networks, peer-to-peer relationships, and social support systems around Mpox prevention. At the community level, we examined participants’ experiences with external Mpox-related gay, bisexual, and transgender community stigma and discrimination. Next, institutional- and policy-level assessments explored multilevel factors that impacted Mpox vaccine implementation and uptake. To that end, throughout each level of the model, we employed an intersectional perspective to understand how participants’ race, sexual orientation, and socioeconomic status intersect with power structures to influence their perceptions about access to the Mpox vaccine.

### Procedures

Study participants were recruited using purposeful sampling procedures and through peer referrals between August 2021 to October 2022 [57]. We utilized purposeful sampling techniques as the primary method for recruitment due to the nature of the topics under investigation, and the study was limited to only BLSMM. Purposeful sampling allowed us to maximize recruitment efforts and facilitated the selection of study participants with information-rich narratives relevant to the topic phenomenon under investigation [24, 65, 71]. The study’s promotional materials were also sent to local community organizations in California and New York and posted on internet-based websites such as Facebook, Instagram, and Twitter. Other study participants were recruited through direct contact with study team members during interactions at social events within the community. Potential participants who expressed interest in the study contacted the study team via email and followed up by telephone or Zoom web conferencing to complete the screening questions and establish eligibility. In order to determine eligibility, a member of the research team confirmed that potential participants were (a) 18 years of age or older, (b) assigned male sex at birth, (c) lived in California or New York at the time of the study, (d) identify as Black or Latino, and (e) had anal or oral sex with a man within the last year. Participants meeting all study eligibility criteria were consented and invited to complete an online demographic and behavior survey administered via *Qualtrics* (an online survey tool). They were then scheduled for their semi-structured, in-depth individual interview. All study participants were interviewed via Zoom. All study interviews were audio-recorded and then transcribed verbatim. Participant interviews lasted between 75 and 90 min. Each participant received a $50 electronic gift card after completing all study-related activities.

Overall, 63 potential participants initially contacted the study team to indicate their willingness to participate in the study. However, of those individuals, only 44 agreed to complete all study-related activities (demographic survey and in-depth individual interview). Of the 44 participants who agreed to participate in the study, only 41 completed the demographic and behavioral survey and the individual interview. Three participants were lost to follow-up. While all participants were assigned male sex at birth, most participants identified as male (*n* = 41) and non-binary or female (*n* = 3). Participants also identified as gay (*n* = 27), bisexual (*n* = 5), heterosexual (*n* = 1), and queer (*n* = 3). Several participants did not record a response for their sexual orientation or reported “other” (*n* = 8). Study participants were from California (*n* = 23) or New York (*n* = 21). Racially, participants identified themselves as Black or of African heritage (*n* = 32) or Latino (*n* = 11). One person did not list their race. Levels of education varied across the sample, with the majority of the sample having an undergraduate (*n* = 11) or graduate education (*n* = 17). Employment status was also mixed, with half the sample reporting employment (*n* = 22) and the remaining either unemployed or listed others (*n* = 19). A total of four persons did not list their employment status.

### Data Collection and Analysis

Individual, semi-structured interviews and a brief demographic and behavior survey were the two primary sources of data in the present study [72]. Each participant was sent a personalized email invitation to complete the study demographic and behavior survey. The survey consisted of close-ended questions that probed their race/ethnicity, education, income, sexual behaviors, employment status, sexual orientation, and gender identity. The study interview guide, informed by the SEM and intersectionality, consisting of open-ended questions, was first developed in consultation with key stakeholders in the community to explore BLSMM’s experiences with accessing HIV prevention and care services and mental health and social support services during the COVID-19 pandemic. However, based on the emergence of the Mpox epidemic during the COVID-19 pandemic, the interview guide was later modified to accommodate Mpox-related content. Although the interview guide was developed to provide a systematic sequencing of the study topic areas, members of the research team were flexible and allowed for digressions away from more sensitive topics or other topics that may be of interest to the participant [50]. All interviews were conducted in English. A secondary data source was the completion of reflexive memos at the end of each interview. The reflexive memos were used to record the observations and impressions of the researcher and study participants. Reflexive memos were either audio-recorded or written in the journal of a research team member, which later aided in the analysis of the data [31, 69, 78].

Qualitative data management and analysis were conducted using the qualitative analysis computer software ATLAS.ti (Version 23.4). Each in-depth interview was audio-recorded and transcribed verbatim by a University of California, San Francisco IRB-approved transcriptionist. All de-identified transcripts were later uploaded into ATLAS.ti for data storage and management. An interpretive phenomenological analysis approach using a collaborative coding process was used to identify themes. All team members were trained by the lead author, an expert in qualitative research, on qualitative research design and analysis before the start of the process. A hallmark of the analysis process in interpretive phenomenological studies is to evaluate the data to identify a paradigm case, a strong instance of a pattern of narrative that hangs together or exemplar quotes. These smaller narratives add nuance and variability to patterns observed that elucidate the phenomena under investigation [24, 57]. In addition, another feature of interpretive phenomenological studies is the process of horizontalization. In this study, hornizontalization was performed by giving equal value and importance to each of the narratives and coding them with descriptive labels [53, 56].

**Fig. 1:**
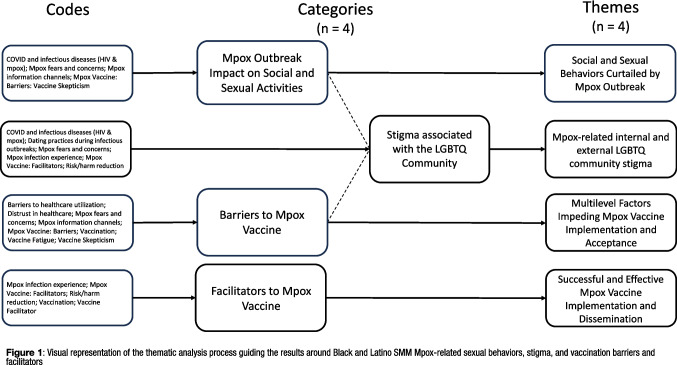
Visual representation of the thematic analysis process guiding the results around Black and Latino SMM Mpox-related sexual behaviors, stigma, and vaccination barriers and facilitators

Data analysis was executed in a stepwise process by the research team consisting of the principal investigator, co-investigator, project manager, and four research assistants. Since codes were not created a prior, each interview transcript was reviewed and coded by members of the research team using an open coding technique to capture large passages of meaningful text throughout the analysis process [12]. Initial codes (*n* = 143) derived from the open coding process were reviewed, compared with each other for similarities, and concluded with the collapsing or elimination of duplicate codes, yielding a final codebook consisting of 81 codes. The next step in the data analysis process was to review the narratives attached to a code and write code summaries, reflecting the collective narratives within that code. The team then reviewed and evaluated code summaries for further code alignment during our weekly research team meetings. Codes with similar topic areas were clustered together and later represented as categories [65]. For example, codes labeled dating practices during infectious disease outbreaks, Mpox fears and concerns, and Mpox infection experience were clustered together into a single category labeled stigma associated with the LGBTQ community. Each category was evaluated for similarities for further refinement. Data analysis continued until saturation was achieved [65]. Saturation was achieved when no new insights or themes emerged, indicating that further data collection and analysis was unnecessary. The final step in the analysis process concluded with the development of themes. The team collectively evaluated code categories to identify one or more common themes or subthemes across categories. Once themes were identified across categories, thematic statements were written to reflect the common themes across the different categories (see Fig. 1 for a visual representation of the thematic analysis process guiding the results around BLSMM Mpox-related sexual behaviors, stigma, and vaccination barriers and facilitators).

### Research Team Positionality

The study was conducted by a diverse team of researchers and student researcher assistants (e.g., medical and nurse practitioners). The research team consisted of gender and sexually diverse people. The study team also consisted of those who identify as African Americans, Afro-Caribbean, White, Latino, and Middle Eastern. The lead researcher and first author of this manuscript identifies as an Afro-Caribbean, cisgender man with extensive clinical and research experience working with sexual and gender minorities in the United States and the Caribbean. His own clinical and lived experiences underscored his interest in initiating this study. Throughout data collection and analysis, the research team met frequently to reflect on and examine our positionality and other experiences that may impact the collection and interpretation of the data [25]. Our experiences include (a) awareness and knowledge of issues affecting BLSMM and other sexual and gender minorities during the Mpox epidemic of 2022 and (b) first-hand experiences as a Black or Latino sexual or gender minority in obtaining vaccines for Mpox.

## Results

### Demographic Characteristics of Study Participants

The demographic and behavioral characteristics of the sample are detailed in Table 1. A total of 44 people agreed to participate in the study. However, only 41 individuals completed all study-related activities. The mean age of participants was 42 (range 19–65). Black/African Americans comprised 73% of the sample, while Latino men accounted for the remaining 25%. The majority of participants had either completed undergraduate or graduate education (64%). A significant proportion of the sample identified as male (93%) and 73% identified either as gay or bisexual.

**Table 1:** Sociodemographic characteristics of the sample (*N* = 44)

	*n*	%
Age (*mean* = *42.43 years)*		
19–29 years	9	20.5
31–39 years	12	27.2
40–49 years	11	25.0
51–56 years	6	13.7
60–65 years	5	11.3
Missing	1	2.3
Sex assigned at birth		
Male	44	100
Gender		
Male	41	93.2
Female	1	2.3
Non-binary/third gender	2	4.5
Sexual orientation		
Gay	27	61.4
Bisexual	5	11.4
Heterosexual or straight	1	2.3
Queer	3	6.8
Other^1^	4	9.1
Missing	4	9.1
Race/ethnicity		
Black or African American	32	72.7
Hispanic or Latinx	11	25.0
Missing	1	2.3
Site		
California	23	52.3
New York	21	47.7
Levels of education completed		
High school	8	18.2
Junior college or vocational school	3	6.8
Undergraduate school	11	25.0
Graduate or professional school	17	38.6
Other^2^	1	2.3
Missing	4	9.1
Employment situation		
Unemployed	9	20.5
Employed	21	47.7
Other^3^	10	22.7
Missing	4	9.1

^1^*Note.* Other: Pan, Aego-, and Demisexual; Demisexual; Same-Gender Loving

^2^*Note*. Other: GED

^3^*Note*. Other: Retirement; SSA; General Relief and Food Stamps; Internship; SSDI; SSI; Student

### Central Themes

The findings in this report reflect the four main themes that emerged from the data analysis. These themes represent participants’ experiences with curtailing their sexual behaviors and practices during the Mpox outbreak of 2022 and the barriers and facilitators that guided or inhibited their access and uptake of vaccines for Mpox. The four identified themes were (a) social and sexual behaviors curtailed by the Mpox outbreak; (b) Mpox-related internal and external LGBTQ community stigma; (c) multilevel factors impeding Mpox vaccine implementation and acceptance; and (d) successful and effective Mpox vaccine implementation and dissemination. In the sections below, all four themes are explained in detail and accompanied by narrative exemplar quotes to illuminate the study findings.

### Social and Sexual Behaviors Curtailed by Mpox Outbreak

For many BLSSM in the study, the Mpox epidemic, which cooccurred at the height of the COVID-19 pandemic and the ongoing HIV crisis, brought into focus the risk of contracting another infectious disease. Participants were uniquely aware of how the Mpox virus was impacting sexual minority communities across the United States, but more specifically, the BLSSM community. For some participants, the COVID-19 pandemic and the Mpox epidemic both paralleled the HIV/AIDS pandemic in terms of their disproportionate impact on SMM.

[I avoided getting Mpox] the same way I did 20, 30 years ago when HIV first came out. I’m avoiding people. And you can only get through my defenses if I know you, first and foremost. No strangers, period. And I need to know some things about you. That’s how you can get through my defenses. And you can’t even get naked in my bed until I know what’s going on with you medically. [Black, New York, 54 years old]

So there was no romance or anything sexual as it got more serious, because I was just like, again, I don’t want another thing. I ain’t even got the vaccines. I don’t even know what’s going on. But with men in my life, I will talk to them about it, like other Black men in the Bay Area. But romantically, no. [Black, California, 36 years old]

I think I was actually at one of my appointments and my provider was talking to me about really the uptick in monkeypox cases that were coming into the community, that they were seeing people at my healthcare center. Whenever I get that kind of information, I try to respond accordingly. There was definitely something that shifted, any practices that we had as a couple or additional partners or whatever that we sort of said, you know, we got to make sure we’re vaccinated first and then go on from there. [Black, New York, 40 years old]

Their awareness of the pervasiveness of the Mpox outbreak resulted in a massive shift in lifestyle to the point where social and sexual interactions were curtailed due to fear of exposure to the virus. For example, one participant from California states, “Oh, honey, I do not want that… When that came out, I definitely stayed inside the house.” Many participants also reported abstaining from sexual activity for an extended period as a form of risk reduction strategy against the virus. Another participant from California stated, “There was no romance or anything sexual… I didn’t want another thing. I ain’t even got the vaccines yet….” Others avoided frequenting LGBTQ social spaces or stayed at home as another way of avoiding Mpox.

Well, that was a time when I decided not to go out. So it didn’t have a big impact on me, and plus, because I had like two friends that got it. So I got scared. And we had like some patients that had it, even kids. Because I work for pediatrics, so and it was some kids with monkeypox. You see how painful it is. That’s when you realize that, oh my God, I don’t want that. I don’t want to be positive with that. [Latino, California, 44 years old]

### Mpox-Related Internal and External LGBTQ Community Stigma

Stigmatization around the Mpox outbreak impacted BLSMM communities both internally, intrapersonal conversations and perceptions within the community, and externally, perceptions and language used to describe BLSMM communities. For some older participants, Mpox-related stigma reminded them of the AIDS crisis when it first emerged in the 1980s. Most participants commented that public health messages around the outbreak and the communities being affected, which were predominantly gay and bisexual men, contributed significantly to the stigma that people within the community experienced. Participants narratives revealed that internalized Mpox stigma manifested in the form of fear of contracting Mpox from other gay and bisexual men, LGBTQ community perception around who was at risk, and the potential for permanent scarring from the Mpox lesions or from the subdermal administration of the Mpox vaccine on the participant’s forearm. External stigma emerged because of health departments’ stringent eligibility criteria for the vaccine, public perception around who is at risk, and the media portrayal of the demographic of those who were disproportionately impacted. The information permeating throughout BLSMM communities made some participants less concerned about acquiring Mpox because, at the time, the media were reporting mostly White gay men of a certain age as being at the highest risk of Mpox.

I was like oh, it’s just another thing on top of another thing that gives the other side leverage to help spew their ignorance almost, right?... I look at it [Mpox] as something they’re going to use against us. AIDS was one, and now monkeypox. It’s like, oh, he must be gay; he’s got monkeypox. Now, you feel like what the Nazis did during World War II with the branded star identifying the Jews. It’s like a label. For me… I know they’re going to weaponize this. [Black, New York, 41 years old]

I remember when it was first on the scene, I was watching Channel 2 news…the 10:00pm News and I was dozing off, and they were talking about it [Mpox]. What woke me back up was when they said it’s a bisexual and gay men’s disease. I just said BS and turned the TV off. I’m like, they’re always trying to throw stuff on us. But then the next morning when I went [into work], it was this whole thing [being talked about]. We were reading about it, talking about it, and so I got upset. I’m like, so how does this virus have a gender, you know what I’m saying? So women can’t, if this is skin to skin contact, like your skin has no orientation, you know what I’m saying? So by me being gay, if I go touch my heterosexual sister and I have monkeypox, wouldn’t she contract it too? So it was like, here we go again. It brought up a lot of the HIV stuff. So now, my understanding is that there was just more of a higher prevalence when they were getting the numbers. The data was showing that it was more prevalent in gay and bisexual men. Okay, so say that. Don’t just be on the news talking about this is a bisexual and gay disease. [Black, California, 39 years old]

Public health messaging around the criteria for Mpox vaccination was also identified by participants as contributors to Mpox-related stigma within their communities. At the peak of the outbreak and with the limited availability of vaccines, the CDC and public health departments created stringent criteria for Mpox vaccination. Many participants in the study did not associate themselves with those criteria and refused to tell untruths to access the Mpox vaccine.

Additionally, several participants also worked for their local health departments and were part of the Mpox response. They saw first-hand how the information around the virus and the lack of access to the vaccine affected them individually and their communities. A participant in the study who worked in the clinical setting suggested that the public health system in the country had gotten the Mpox messaging completely wrong. He stated:

I feel like it’s how it’s been labeled. It reminded me of the AIDS crisis when it was first considered as a gay illness. One of the reasons why I haven’t taken the vaccine is because of the [screening] questions. I am not going to lie, and I would never tell my patients to lie. When I found out my husband got the vaccine, I was like oh, ‘have you had multiple sex partners in the last two weeks?’ Which was a joke, of course; well, I hope it is. But two of the questions asked if you have had anonymous sex or multiple sex partners within one or two weeks, and I have not. Until they change those criteria, and everyone is included regardless of whether they’re having anonymous or promiscuous sex, then I will take it. But for now, I’m not going to lie on those questions just because I want it. It should not be, and then you’re using those data to say oh, these people are having multiple sex partners. You shouldn’t force people to do what’s wrong, and I’m a firm believer in that. [Black, New York, 38 years old]

Similarly, another participant who worked in the clinical setting commented that even within his own clinical setting, among his colleagues, there was also Mpox-related stigma toward the LGBTQ community. He stated:

People were just saying, because a lot of people at my job were saying but oh, that only happens to you guys, to gay people. I’m like, no. Come on. Nobody’s exempt of anything. You know? [Latino, California, 44 years old]

### Multilevel Factors Impeding Mpox Vaccine Implementation and Acceptance

There were several multilevel factors that impeded Mpox vaccine implementation, access, and uptake among study participants. For some participants, distrust in the healthcare system, poor health communication about Mpox risk, vaccine dosage skepticism, and vaccine fatigue due to the need for multiple vaccinations since the start of the COVID-19 pandemic influenced their acceptance and uptake of the Mpox vaccine. One participant who is living with HIV talked more extensively about the type of vaccine fatigue he experienced. For this participant, living with HIV meant that he would have needed multiple dosages of the COVID-19 vaccines as well as the annual influenza and pneumococcal vaccines because he was immunocompromised. “I’m already taking HIV medicines. I got two vaccines [COVID-19]. I don’t want more stuff [in my body]. I feel it’ll do something to my immune system. I just don’t trust it.” Those participants who expressed skepticism of the Mpox vaccine reported that they were hesitant to get vaccinated “because the monkeypox vaccine is new. It hasn’t gone through trial and error yet. I just don’t trust it.” Distrust emerged as a reoccurring theme for deciding against the vaccine. For others who were interested in receiving the vaccine, limited availability, supply chain issues, long lines with extended wait times outdoors during the hot summer months, and the scarring associated with vaccination were all described as vaccine implementation barriers.

I think initially I had tried to get a vaccine for it [Mpox]. But I’m not like sexually active very much or hardly these days. I didn’t see myself as high risk. But even though I was eligible to get it because of how I answered the [screening] questions. I didn’t get it because I think they were giving them out at [x hospital], but they ran out, and there was like a huge line, and I was like, I’m not going to wait in that line. [Black, California, 42 years old]

Several participants also complained about encountering glitches with state and local websites when attempting to schedule their Mpox vaccination appointment. In addition, some participants commented that when the virus was affecting mostly White and Asian gay men, they were able to receive the full dose of the vaccine, which was later reduced in order to vaccinate more people. They believe that because the demographic of the people who were now significantly affected shifted, mostly Black and Latino gay men, there were new recommendations around splitting the dosage of the medication. Some participants were of the belief that the difference in the dosing was racially motivated, and as a result, they did not trust the vaccine. Others commented on the skin condition that came from receiving the intradermal compared to the intermuscular dose.

I couldn’t sign up because the [website] crashed. There were so many people trying to sign up. After several weeks of investigation about how the rollout of the monkeypox vaccine was, it was discovered or the data revealed that White gay men were signing up disproportionately compared to other racial groups. And that even when the sites, the vaccine sites were in communities where White gay men did not live, they were going up there to get the vaccine, where the vaccine was intended for the people who lived in those communities. [Black, New York, 60 years old]

So, one where it leaves a little bubble. Now they’ve been doing the subdural [subcutaneous]. I think that is problematic for some people because it leaves a mark. It is, I think some people, we’ve heard that some people see it as, when they get it, some folks are saying like okay look, I’ve got my vaccination, and its proof that I’ve got my vaccination because I have this mark. There are others who find the mark to be unsightly and as a permanent stigma. [Black, New York, 52 years old]

### Successful and Effective Mpox Vaccine Implementation and Dissemination

There were several effective strategies to vaccine implementation and dissemination that proved to be successful for the men in our study. Participants commented that their trusted medical provider recommendations and the incorporation of vaccine awareness by community leaders were two successful implementation strategies that led them to get vaccinated against the virus. Similarly, supplying community-based clinics and organizations that predominantly serve BLSMM as well as LGBTQ-themed community events (e.g., pride) were viewed as common vaccine access points for participants. Other motivations for receiving the Mpox vaccine include knowing or seeing someone (i.e., friends or acquaintances) with skin lesions who were infected, their employment with the public health department or an organization that served the LGBTQ community, planned travels during the summer and not wanting to come in contact with an infected person at a social event. Additionally, the diversity in the staffing at local vaccination sites, the availability of vaccines at local churches, and having community members build community awareness were all believed to be motivators for vaccination by study participants.

I got information from somebody who knew somebody, oh, well this church in Harlem is vaccinating people. Come to the church and you can get vaccinated and that’s what I did. You didn’t even have to sign up. Just go there. This is the time. I went there and there was hardly anybody in the church to get vaccinated. Like I said, the church was in Harlem, but it was because I knew someone who knew someone who posted it on Facebook. It was because I was connected that I knew about this and I was able to get vaccinated, my initial vaccination. [Black, New York, 60 years old]

I was more inclined to get that one [Mpox instead of the COVID-19 vaccine] because I didn’t want bumps on my face. I’m going to be fully transparent. It definitely was a level of vanity involved in my motivation to getting that. [Black, California, 35 years old]

I’ve gotten vaccinated. I’m glad that my community members have been vocal and organizing around increasing our awareness and access to these resources and information. They’ve been a direct support to me in regard to me having gotten updated… And me looking to get my appointment set up and get this shit taken care of. I’ve gotten my monkeypox vaccine, both the first and second doses. [Black, New York, 42 years old]

## Discussion

The narratives presented throughout this manuscript highlighted how participants curtailed their social interactions and sexual behaviors during the Mpox outbreak of 2022–2023. The findings also shed light on the experience of Mpox-related stigma that impacted the community. The breadth and depth of Mpox-related stigma was experienced internally within the BLSMM community and externally as part of the larger society. Additionally, the findings in this report also illuminated multilevel factors that impacted BLSMM access to the Mpox vaccine, with institutional and structural factors identified as barriers to successful Mpox vaccine implementation and uptake. Moreover, despite the barriers that many participants encountered in accessing the Mpox vaccine, their collective narratives explained successful and effective Mpox vaccine implementation and dissemination. Our methodological approach, which was informed by our application of the socioecological model and an intersectional framework, informed our ability to understand how the Mpox phenomenon and its convergence with both the COVID-19 and HIV pandemic impacted the lives of BLSSM in our study.

The men in our study reported that their social and sexual behaviors were curtailed as one form of their risk reduction strategy to abet potential Mpox infection. Specifically, many reported abstaining from sexual activity with casual sexual partners for an extended period of time or until they were able to receive a vaccine against the virus. This risk reduction strategy is not unique to the BLSMM that participated in our study. Behaviors such as avoidance of unprotected anal sex, sex in gay-related venues, and open relationships between men and their primary partners were observed among other SMM in the United States and globally [1, 5, 42, 66]. Additionally, one particular study found that most sexually active participants adopted at least one sexual risk reduction behavior (i.e., avoidance of anal sex and sexual with anonymous partners) due to Mpox [66], which is also similar to the men in our study who were less likely to be sexually active yet reported risk reduction behaviors such as avoiding sex with anonymous partners (i.e., “no hookups”). This is not surprising as most participants reported, based on the information from the media and their friends, that Mpox was transmitted through sexual contact; therefore, avoiding sex was seen as the best strategy [54]. Their knowledge of the mode of transmission for Mpox is supported by a growing body of evidence that determined that sexual transmission was the primary mode of transmission for the Mpox virus and recommendations that followed to label it as a sexually transmitted disease [3].

Mpox-related stigma emerged as a factor that impacted our participants both internally and externally. For many participants, media reports of the communities being affected initially made them believe that they were not at risk for Mpox. According to news reports and epidemiological data at the time, White and Asian cisgender gay men were more likely to be affected by Mpox [49], leaving many minoritized and marginalized communities behind in terms of the public health response. Additional public health messaging around risk factors for Mpox, such as having multiple sexual partnerships, being sexually active at commercial sex venues, sex in exchange for money or drugs, and living with HIV were all identified as factors that contributed to external Mpox stigma by participants in our study. Many of our participants compared the stigma of Mpox to HIV, especially during the early years of the HIV pandemic. There are several studies which have emerged that drew these same parallels between Mpox and HIV stigma and provided insights for further public health research, especially those focusing on stigma and discrimination [6, 51, 52]. Internalized community stigma among study participants was observed in the form of fear of contracting Mpox, permanent scarring from the Mpox lesions, or scarring after receiving the vaccine. Some of these concerns were also observed in similar populations of SMM in the US and offer an understanding as to how public health can best reduce new Mpox infections among this population [51, 61, 80]

The effort to vaccinate gay and bisexual men against Mpox met several challenges in the US. Some of these challenges directly resulted from the vaccination criteria and risk profile set by the CDC and other health departments [1, 4, 60]. For example, a person was eligible for the vaccine if they identified as SMM who had sex in the past 6 months, had multiple partners, was diagnosed with an STI, had sex at a commercial venue, had sex in exchange for money, or had sex with a person living with HIV. Many of the men in our study did not receive the vaccine at the time of the vaccine rollout because they believed they did not meet those stringent criteria. However, once the epidemiological data shifted, demonstrating that Black and Latino SMM were now disproportionately impacted, the availability of vaccines and changes in dosing recommendations led to a pervasive sense of distrust of public health and the healthcare infrastructure [26, 76]. Those participants interested in receiving the vaccine cited limited vaccine availability due to supply chain issues, long wait times, and the scarring from the vaccine as barriers to vaccination. In addition, the recommendation to change the dosage and site of administration of the vaccine (intradermal to subcutaneous) also contributed to distrust among study participants. Due to the need to vaccinate more people, the CDC recommended a lower dose of the Mpox vaccine, which they proclaimed safe and generated the same level of protection against the virus as the standard regime [20]. However, those recommendations were not well received by study participants, which they thought was racially motivated, and provided some explanations for the low vaccination rates among BLSMM communities [4, 61, 80]. Moreover, these findings are in line with the emerging literature, which demonstrated that BLSMM were disadvantaged in vaccine access and uptake, while White individuals were more advantaged in Mpox vaccine access and uptake [49, 61, 80]. The present study expanded the literature by contextualizing these disparities with vivid narratives highlighting the Mpox-related experiences of BLSMM.

While several factors impeded vaccine access and acceptance among BLSMM in our study, there were several factors that were identified that have increased uptake among study participants. Some of those factors that increased vaccine uptake included trust in their medical providers, information from community leaders, and knowing someone in their social network were all factors that increased vaccine uptake. Moreover, eliminating barriers to accessing the vaccine by making them available at local community events such as LGBTQ pride events, community clinics, and organizations that serve the community were also promotors of vaccination. In a study conducted in the Washington, D.C. metropolitan area through a community-based organization (CBOs) that serves Black SMM, vaccination acceptance and uptake were high, underscoring the need for partnerships with CBOs with a direct relationship with the community [61]. In addition to the study by Ogunbajo et al., there were several other studies that described successful opportunities for increasing Mpox vaccine uptake among this population [19, 26, 38]. It is also worth noting that several of the participants in our study also worked in public health or their local health departments. Their experiences with Mpox vaccine barriers were not different from those of other participants. This highlights that their area of employment was not protective against any of the barriers mentioned above, bringing into focus the experiences of those with intersectional identities who experience health disparities.

The application of both the social-ecological model as well as intersectionality as a framework to better understand Mpox-related experiences provided an opportunity to understand the unique experiences of BLSMM, a group that is marginalized and experiences profound discrimination (i.e., racism, homophobia, and HIV stigma). The SEM model and intersectional framework provided a lens to identify and explore the overlapping impact of social identities (i.e., race, sexual orientation, and socioeconomic status) and other structural factors (i.e., access to health care and Mpox vaccine) on BLSMM’s Mpox-related sex behaviors, stigma, and vaccine uptake. Intersectional framework, rooted in Black feminist pedagogy and praxis, guided our analysis and explained how racism and other drivers of discrimination exacerbated Mpox disparities among BLSMM in our study. Our findings are similar to that of the COVID-19 literature on vaccine uptake among Black and Latino communities where racism, discrimination, distrust, and limited access to the COVID-19 vaccine were all identified as barriers to vaccine [39, 59, 70, 82]. Moreover, the disparities observed with BLSMM’s limited access to the Mpox vaccine were profoundly similar to their experiences with accessing preexposure prophylaxis (PrEP) for the prevention of HIV, when compared to their White counterparts [40, 43]. Since PrEP first became available in 2014 [36], studies have shown that PrEP uptake among BLSMM has remained dangerously low, with social determinants of health and racism as drivers of these disparities [8, 43, 45]. An interesting observation made in this study is that educational attainment, with a majority (63%) of participants reporting having an undergraduate education or higher, did not increase BLSMM access to the vaccine. This observation, along with additional evidence from the literature, suggests race and other determinants of health impacted vaccine uptake among BLSMM [15]. Finally, with the application of SEM and intersectionality, at the individual, interpersonal, and community levels, stigma emerged as a significant barrier to Mpox vaccine uptake among BLSMM. This finding broadens our knowledge of the role of stigma as a barrier to access to PrEP uptake among this population and provides an opportunity for us to apply that knowledge to increase Mpox vaccine uptake among BLSMM [29, 35, 52, 81].

These findings have implications for Mpox-related advocacy, policy, and further research. First, we found that regardless of risk, BLSMM reduced their sexual behaviors and limited their social gatherings as two risk reduction strategies against potential Mpox infection. This implied that BLSMM adhered to public health recommendations even though they were at low risk. Second, Mpox-related stigma, which manifested both externally and internally, affected the community and contributed to low vaccination uptake among this population. In addition, public health messaging around those who were at risk negatively influenced the community as they further contributed to the stigma towards those with the virus. This suggests the need to develop culturally tailored messages that respect the community as well as interventions to reduce stigma among SMM living with or at risk for HIV that go beyond addressing Mpox-related stigma. Third, this research underscores the importance of eliminating barriers to vaccination and expanding vaccine access to all communities, regardless of race, stigmatizing risk profiles, or socioeconomic characteristics. The findings also suggest that efforts to eliminate structural barriers and promote vaccine uptake must be conceived ahead of time and not after those disparities have widened. Finally, our study highlights the complexity of the experience of Mpox as it is experienced by BLSMM in our study. These experiences are complex and require further research using quantitative or mixed methodology to better understand how Mpox was experienced and understood.

### Limitations

The study has several limitations. First, we recruited study participants through purposeful sampling and peer referrals in order to reach the target population. Although study participants are not representative of the experiences of all racialized sexual minority men, the perceptions and experiences shared in this interpretive phenomenological qualitative study have relevance and applicability beyond those BLSMM who participated in the study. Second, the study sample consisted of mostly BSMM, with LSMM comprising less than 30% of the sample. Thus, the narratives and experiences of LSMM may not be fully understood. Therefore, additional studies are needed to further understand their experiences. Third, study participants were predominantly from two urban areas along the western and eastern coast of the United States; therefore, the experiences of those BLSMM who live in rural or southern parts of the country were not represented in the study. We suspect that the Mpox-related experiences of those living in rural areas of both New York, California, and other southern states might be different and that their access to the vaccine might also be limited as most of the vaccination sites were in predominantly urban White gay neighborhoods. Fourth, while we did have age diversity among study participants, those with diverse gender identities were less represented as the focus was on BLSSM. Fifth, not all participants who were contacted participated in the study, and three participants who initially agreed to participate did not complete all study-related activities. Therefore, those who participated were more eager to share their experiences. Finally, despite these limitations, we assert that our interpretations of the findings in this study are a significant contribution to the growing literature around these experiences and are only limited to this sample of Black and Latino MSM living in California and New York.

## Conclusions

As the Mpox virus continues its devastating impact across the globe with the emergence of different mutations, there is a need to increase access and uptake of the vaccine among groups that are disproportionately impacted by the virus. BLSMM in the United States, especially those living with HIV, continue to experience disparities in Mpox infection and disproportionate access and uptake of the vaccine. However, public health and the healthcare system have continued to leave these groups behind, driving more distrust of these systems within the community. Therefore, sustained equity-based strategies, such as culturally tailored prevention messages and expanded vaccine services to reach racial and sexual minority groups, are desperately needed to eliminate all disparities with future Mpox and other disease outbreaks. Additionally, stigma, which is a driver of low engagement in Mpox and HIV prevention and care, can be eliminated with sex-positive harm reduction messaging as opposed to abstinence-only prevention messages. Finally, partnerships between CBOs and public health agencies can facilitate quick and effective dissemination of community-inspired interventions to increase access and uptake of Mpox vaccines.

## References

[1] Abara WE, Sullivan P, Carpino T, Sanchez T, Atkins K, Delaney K, Edwards OW, Hannah M, Baral S, Ogale Y, Galloway E, Lansky A. Characteristics of Mpox vaccine recipients among a sample of men who have sex with men with presumed exposure to Mpox. Sex Transm Dis. 2023;50(7):458–61. 10.1097/OLQ.0000000000001800. 36940183 10.1097/OLQ.0000000000001800 PMC10330397

[2] Aldred B, Scott JY, Aldredge A, Gromer DJ, Anderson AM, Cartwright EJ, Colasanti JA, Hall B, Jacob JT, Kalapila A, Kandiah S, Kelley CF, Lyles RH, Marconi VC, Nguyen ML, Rebolledo PA, Sheth AN, Szabo B, Titanji BK, Cantos VD. Associations between HIV and severe Mpox in an Atlanta cohort. J Infect Dis. 2023. 10.1093/infdis/jiad505 10.1093/infdis/jiad505 38001044

[3] Allan-Blitz LT, Gandhi M, Adamson P, Park I, Bolan G, Klausner JD. A position statement on Mpox as a sexually transmitted disease. Clin Infect Dis ®. 2022;76(8):1508–20. 10.1093/cid/ciac960. 10.1093/cid/ciac960 PMC10110265 36546646

[4] Allan-Blitz LT, Khan T, Elangovan K, Smith K, Multani A, Mayer KH. Addressing mpox at a frontline community health center: lessons for the next outbreak. Public Health Rep. 2024;139(3):294–300. 10.1177/00333549231201682. 37846528 10.1177/00333549231201682 PMC11037218

[5] Amer F, Khalifa HES, Elahmady M, ElBadawy NE, Zahran WA, Abdelnasser M, Rodríguez-Morales AJ, Wegdan AA, Tash RME. Mpox: risks and approaches to prevention. J Infect Public Health. 2023;16(6):901–10. 10.1016/J.JIPH.2023.04.001. 37062165 10.1016/j.jiph.2023.04.001 PMC10074767

[6] Ann S, Id S, Id MG, Lovelace S, Id JJ, Choate C, Guerin J, Weinstein W, Taylor G. Survey of pain and stigma experiences in people diagnosed with mpox in Baltimore, Maryland during 2022 global outbreak. PLoS ONE. 2024;5(19). 10.1371/journal.pone.0299587 10.1371/journal.pone.0299587 PMC11108198 38771788

[7] Banuet-Martinez M, Yang Y, Jafari B, Kaur A, Butt ZA, Chen HH, Yanushkevich S, Moyles IR, Heffernan JM, Korosec CS. Monkeypox: a review of epidemiological modelling studies and how modelling has led to mechanistic insight. In Epidemiology and Infection (Vol. 151). Cambridge University Press. 2023. 10.1017/S0950268823000791 10.1017/S0950268823000791 PMC10468816 37218612

[8] Beltran RM, Holloway IW, Hong C, Miyashita A, Cordero L, Wu E, Burris K, Frew PM. Social determinants of disease: HIV and COVID-19 experiences. In Current HIV/AIDS Reports 2022;19(1): 101–112. Springer. 10.1007/s11904-021-00595-6 10.1007/s11904-021-00595-6 PMC8808274 35107810

[9] Birch L, Bindert A, Macias S, Luo E, Nwanah P, Green N, Stamps J, Crooks N, Singer RM, Johnson R, Singer RB. When stigma, disclosure, and access to care collide: an ethical reflection of Mpox vaccination outreach. Public Health Rep. 2024;139(3):379–84. 10.1177/00333549231201617. 37846098 10.1177/00333549231201617 PMC11037228

[10] Bowleg L. The problem with the phrase women and minorities: Intersectionality-an important theoretical framework for public health. Am J Public Health. 2012;102(7):1267–73. 22594719 10.2105/AJPH.2012.300750 PMC3477987

[11] Bowleg L. We’re not all in this together: On COVID-19, intersectionality, and structural inequality. Am J Public Health. 2020;110(7):917–8. 10.2105/AJPH.2020.305766. 32463703 10.2105/AJPH.2020.305766 PMC7287552

[12] Braun V, Clarke V. Using thematic analysis in psychology. Qual Res Psychol. 2006;3(2):77–101.

[13] Brihn A, Yeganeh N, Kulkarni S, Moir O, Madrid S, Perez M, Singhal R, Kim AA. Countering Mpox vaccination disparities in Los Angeles County, California, May–December 2022. Am J Public Health. 2023;113(12):1258–62. 10.2105/AJPH.2023.307409. 37733994 10.2105/AJPH.2023.307409 PMC10632831

[14] Brooks KA, Neptune NS, Mattox DE. Otolaryngologic manifestations of Mpox: the Atlanta outbreak. Acta Otolaryngol. 2023;143(3):237–41. 10.1080/00016489.2023.2182911. 36896982 10.1080/00016489.2023.2182911

[15] Carpino T, Atkins K, Abara W, Edwards OW, Lansky A, DiNenno E, Hannah M, Delaney K, Murray S, Sanchez T, Baral S. Mpox and vaccine knowledge, beliefs, and sources of trusted information among gay, bisexual, and other men who have sex with men in the United States. AJPM Focus, 2024;100267. 10.1016/j.focus.2024.100267 10.1016/j.focus.2024.100267 PMC11440292 39350798

[16] Carrico S, Zitta J-P, Stevens E, Jenkins R, Mortiboy M, Jenks JD. Mpox vaccination and the role of social vulnerability in Durham County, North Carolina, USA. J Racial Ethnic Health Disparities. 2023. 10.1007/s40615-023-01827-8 10.1007/s40615-023-01827-8 37831364

[17] Centers for Disease Control. Diagnoses, deaths, and prevalence of HIV in the United States and 6 territories and freely associated states, 2022. HIV Surveillance Report. 2024a. https://www.cdc.gov/hiv/data-research/facts-stats/gay-bisexual-men.html

[18] Centers for Disease Control. Behavioral and clinical characteristics of persons with diagnosed HIV infection—medical monitoring project, United States, 2022 cycle (June 2022–May 2023). HIV Surveillance Special Report. 2024b. https://www.cdc.gov/hiv/data-research/facts-stats/gay-bisexual-men.html

[19] Chan ASW. Unveiling racial and ethnic disparities in MPOX virus vaccine distribution and demographic patterns in the United States. Multidisciplinary Digital Publishing Institute. 2023, 10.20944/preprints202311.0275.v1

[20] Cices A, Prasad S, Akselrad M, Sells N, Woods K, Silverberg NB, Camins B. Mpox update: clinical presentation, vaccination guidance, and management. Cutis. 2023;111(4):197–202. 10.12788/cutis.0745. 37289697 10.12788/cutis.0745

[21] Collins P. *Black feminist thought: knowledge, consciousness, and the politics of empowerment* (Second). Routledge; 2000.

[22] Coustasse A, Kimble C, Maxik K. COVID-19 and vaccine hesitancy: a challenge the United States must overcome. J Ambul Care Manage. 2021;44(1):71–5. 10.1097/JAC.0000000000000360. 33165121 10.1097/JAC.0000000000000360

[23] Crenshaw KW. Demarginalising the intersection of race and sex: a black feminist critique of anti-discrimination doctrine, feminist theory, and anti-racist politics. Univ Chic Leg Forum. 1989;1(8):139–67. 10.4324/9781315582924-10.

[24] Creswell JW, Hanson WE, Clark Plano VL, Morales A. Qualitative research designs: selection and implementation. Couns Psychol. 2007;35(2):236–64. 10.1177/0011000006287390.

[25] Creswell JW, Plano-Clark VL. Choosing a mixed methods design. Designing and Conducting Mixed Method Research. 2011.

[26] Curtis MG, Davoudpour S, Rodriguez-Ortiz AE, Felt D, French AL, Hosek SG, Phillips G, Serrano PA. Predictors of Mpox vaccine uptake among sexual and gender minority young adults living in Illinois: Unvaccinated vs. double vs. single dose vaccine recipients. Vaccine. 2023;41(27):4002–8. 10.1016/j.vaccine.2023.05.043. 37236817 10.1016/j.vaccine.2023.05.043 PMC10206605

[27] Delaney KP, Sanchez T, Hannah M, Edwards OW, Carpino T, Agnew-Brune C, Renfro K, Kachur R, Carnes N, DiNenno EA, Lansky A, Ethier K, Sullivan P, Baral S, Oster AM. Strategies adopted by gay, bisexual, and other men who have sex with men to prevent monkeypox virus transmission — United States, August 2022. MMWR Recommendations and Reports. 2022;71(35):1126–30. 10.15585/MMWR.MM7135E1. 10.15585/mmwr.mm7135e1 PMC9472779 36048582

[28] Drake RE, Sederer LI, Becker DR, Bond GR. COVID-19, unemployment, and behavioral health conditions: the need for supported employment. Adm Policy Mental Health Mental Health Services Res. 2021;48:388–92. 10.1007/s10488-021-01130-w. 10.1007/s10488-021-01130-w PMC8011768 33791925

[29] Elopre L, Kudroff K, Westfall AO, Overton ET, Mugavero MJ. Brief Report: The right people, right places, and right practices: disparities in PrEP access among African American men, women, and MSM in the deep south. J Acquir Immune Defic Syndr. 2017;74(1):56–9. 10.1097/QAI.0000000000001165. 27552156 10.1097/QAI.0000000000001165 PMC5903558

[30] Essajee NM, Oddo-Moise H, Hagensee ME, Lillis RA, Maffei J, Butler I, Lovett A, Sokol T, Clement ME. Characteristics of Mpox infections in Louisiana in the 2022 outbreak. AIDS Res Human Retroviruses, 2023;39(11). 10.1089/aid.2023.0011 10.1089/aid.2023.0011 PMC10621655 37424520

[31] Finlay L. “Outing” the researcher: the provenance, process, and practice of reflexivity. Qual Health Res. 2002;12(4):531–45. 11939252 10.1177/104973202129120052

[32] Garneau WM, Jones JL, Dashler GM, Kwon N, Hamill MM, Gilliams EA, Rudolph DS, Keruly JC, Klein EY, Wang N-Y, Hansoti B, Gebo KA. Adherence to CDC guidelines for Mpox evaluation: practice patterns across an academic medical system during the 2022 epidemic. Open Forum Infect Dis. 2024;11(9):ofae512. 10.1093/ofid/ofae512. 39323905 10.1093/ofid/ofae512 PMC11422182

[33] Garneau WM, Jones JL, Dashler GM, Mostafa HH, Judson SD, Kwon N, Hamill MM, Gilliams EA, Rudolph DS, Keruly JC, Fall A, Klein EY, Hansoti B, Gebo KA. Risk factors for hospitalization and effect of immunosuppression on clinical outcomes among an urban cohort of patients with Mpox. Open Forum Infect Dis. 2023. 10.1093/ofid/ofad533. 38058459 10.1093/ofid/ofad533 PMC10697423

[34] Golden SD, Earp JAL. Social ecological approaches to individuals and their contexts: twenty years of health education and behavior health promotion interventions. Health Educ Behav. 2012;39(3):364–72. 10.1177/1090198111418634. 22267868 10.1177/1090198111418634

[35] Golub SA. PrEP stigma: implicit and explicit drivers of disparity. Curr HIV/AIDS Rep. 2018;15(2):190–7. 10.1007/s11904-018-0385-0. 29460223 10.1007/s11904-018-0385-0 PMC5884731

[36] Grant R, Lama J, Anderson P, McMahan V, Lui A, Vargas L, Goicochea P, Casapia M, Guanira-Carranza J, Ramirez-Cardich M. Preexposure chemoprophylaxis for HIV prevention in men who have sex with men. TNew England J Med. 2010;363(27):2587. 10.1056/NEJMoa1011205. 10.1056/NEJMoa1011205 PMC3079639 21091279

[37] Grov C, Zohra F, Mirzayi C, Stief M, D’Angelo AB, Dearolf M, Westmoreland DA, Carneiro P, Nash D, Carrico AW. Sexual and gender minorities’ vaccine uptake and behavioral change in response to the Mpox outbreak in the United States: August 2022 through November 2022. Clin Infect Dis. 2024. 10.1093/cid/ciad793. 38262167 10.1093/cid/ciad793

[38] Guilamo-Ramos V, Thimm-Kaiser M, Benzekri A. Community-engaged Mpox vaccination provides lessons for equitable health care in the United States. In Nature Medicine. Nature Research. 2023;29(9):2160–2161. 10.1038/s41591-023-02447-9 10.1038/s41591-023-02447-9 37468666

[39] Harris OO, Perry TE, Johnson JK, Lichtenberg P, Washington T, Kitt B, Shaw M, Keiser S, Tran T, Vest L, Maloof M, Portacolone E. Understanding the concept of trust and other factors related to COVID-19 vaccine intentions among Black/African American older adults prior to vaccine development. SSM - Qualitative Research in Health, 2023;3. 10.1016/j.ssmqr.2023.100230 10.1016/j.ssmqr.2023.100230 PMC9898052 36785539

[40] Harrison SE, Muessig K, Poteat T, Koester K, Vecchio A, Paton M, Miller SJ, Pereira N, Harris O, Myers J, Campbell C, Hightow-Weidman L. Addressing racism’s role in the US HIV epidemic: qualitative findings from three ending the HIV epidemic prevention projects. J Acquir Immune Defic Syndr. 2022;90:S46–55. 10.1097/QAI.0000000000002965. 35703755 10.1097/QAI.0000000000002965 PMC9204779

[41] Hassan R, Wondmeneh S, Gonzalez Jimenez N, Chapman K, Mangla A, Ashley P, Willut C, Lee M, Rhodes T, Gillani S, Copen C, Jackson DA, Waltenburg M, Delaney KP, Miles G, Agnew-Brune C, Oakley LP, Ahmad A, Anthony E, Yang Y. Mpox knowledge, attitudes, and practices among persons presenting for JYNNEOS vaccination - District of Columbia, August to October 2022. Sexually Transmitted Diseases. 2024;51(1):47–53. 10.1097/OLQ.0000000000001893. 37921836 10.1097/OLQ.0000000000001893 PMC11027964

[42] Hazra A, Rusie LK, Wasanwala T, Sachdev N, Guidry T, Tabidze I, Mehta SD. Impact of COVID-19 and Mpox on sexual practices and disease mitigation strategies over time among men who have sex with men affiliated with collective sex venues. Sex Transm Dis. 2024. 10.1097/OLQ.0000000000002063. 39115209 10.1097/OLQ.0000000000002063

[43] Hill M, Smith J, Elimam D, Mustafaa G, Wortley P, Taylor B, Harris O. Ending the HIV epidemic PrEP equity recommendations from a rapid ethnographic assessment of multilevel PrEP use determinants among young Black gay and bisexual men in Atlanta. GA PLoS ONE. 2023;18(3 March):e0283764. 10.1371/journal.pone.0283764. 36996143 10.1371/journal.pone.0283764 PMC10062590

[44] Hughes D, Ai J, Vazirnia P, McLeish T, Krajco C, Moraga R, Quinn K. A qualitative study of Chicago gay men and the Mpox outbreak of 2022 in the context of HIV/AIDS, PrEP, and COVID-19. BMC Infect Dis. 2024;24(1):1174. 10.1186/s12879-024-09491-x. 39425023 10.1186/s12879-024-09491-x PMC11488211

[45] Joudeh L, Harris OO, Johnstone E, Heavner-Sullivan S, Propst SK. “Little Red Flags”: barriers to accessing health care as a sexual or gender minority individual in the rural Southern United States - a qualitative intersectional approach. J Assoc Nurses AIDS Care. 2021;32(4):467–80. 10.1097/JNC.0000000000000271. 33935190 10.1097/JNC.0000000000000271 PMC8238829

[46] Karagoz A, Tombuloglu H, Alsaeed M, Tombuloglu G, AlRubaish AA, Mahmoud A, Smajlović S, Ćordić S, Rabaan AA, Alsuhaimi E. Monkeypox (mpox) virus: classification, origin, transmission, genome organization, antiviral drugs, and molecular diagnosis. In Journal of Infection and Public Health. 2023;16(4):531–541. Elsevier Ltd. 10.1016/j.jiph.2023.02.003. 10.1016/j.jiph.2023.02.003 PMC9908738 36801633

[47] Kiran Kota K, Hong J, Zelaya C, Riser AP, Rodriguez A, Weller DL, Spicknall IH, Kriss JL, Lee F, Boersma P, Hurley E, Hicks P, Wilkins C, Chesson H, Concepción-Acevedo J, Ellington S, Belay E, Mermin J. Racial and ethnic disparities in Mpox cases and vaccination among adult males-United States, May-December 2022. Morbidity and Mortality Weekly Report, 2023a;72(15). https://www.cdc.gov/poxvirus/monkeypox/response/2022/vaccines_data.html 10.15585/mmwr.mm7215a4 PMC10121252 37053122

[48] Kiran Kota K, Hong J, Zelaya C, Riser AP, Rodriguez A, Weller DL, Spicknall IH, Kriss JL, Lee F, Boersma P, Hurley E, Hicks P, Wilkins C, Chesson H, Concepción-Acevedo J, Ellington S, Belay E, Mermin J. Racial and ethnic disparities in Mpox cases and vaccination among adult males-United States, May-December 2022. Morbidity and Mortality Weekly Report, 2023b;72(15), 398–403. https://www.cdc.gov/poxvirus/monkeypox/response/2022/vaccines_data.html 10.15585/mmwr.mm7215a4 PMC10121252 37053122

[49] Krause KD, Lewis K, Scrofani S, Guo TY, Goulbourne D, Halkitis PN. Health behaviors and experiences of LGBTQ + individuals during 2022 Mpox outbreak: findings from the QVax study. J Community Health. 2024. 10.1007/s10900-024-01383-0. 39183233 10.1007/s10900-024-01383-0 PMC11805836

[50] Krueger R, Casey MA. Overview of focus groups. In: Focus groups: a practical guide for applied research. Sage. 2015

[51] Le Forestier J, Skakoon-Sparling S, Page-Gould E, Chasteen A. Supplemental material for experiences of stigma among sexual minority men during the 2022 Global Mpox Outbreak. Psychology of Sexual Orientation and Gender Diversity, 2024;2329–0382. 10.1037/sgd0000739.supp

[52] Logie CH. What can we learn from HIV, COVID-19 and Mpox stigma to guide stigma-informed pandemic preparedness? CH J Int AIDS Soc. 2022;2022:26042. 10.1002/jia2.26042/full. 10.1002/jia2.26042 PMC9721204 36468931

[53] Lopez KA, Willis DG. Descriptive versus interpretive phenomenology: their contributions to nursing knowledge. In Qualitative Health Research, 2024;14(5): 726–735. 10.1177/1049732304263638 10.1177/1049732304263638 15107174

[54] Macgibbon J, Cornelisse VJ, Smith AKJ, Broady TR, Hammoud MA, Bavinton BR, Heath-Paynter D, Vaughan M, Wright EJ, Holt M. Mpox (monkeypox) knowledge, concern, willingness to change behaviour, and seek vaccination: results of a national cross-sectional survey. Sexual Health. 2023;20(5):403–10. 10.1071/SH23047. 37611539 10.1071/SH23047

[55] Mackey K, Ayers CK, Kondo KK, Saha S, Advani SM, Young S, Spencer H, Rusek M, Anderson J, Veazie S, Smith M, Kansagara D. Racial and ethnic disparities in covid-19-related infections, hospitalizations, and deaths a systematic review. Ann Intern Med. 2021;174(3):362–73. 10.7326/M20-6306. 33253040 10.7326/M20-6306 PMC7772883

[56] MacKey S. Phenomenological nursing research: Methodological insights derived from Heidegger’s interpretive phenomenology. Int J Nurs Stud. 2005;42(2):179–86. 10.1016/j.ijnurstu.2004.06.011. 15680616 10.1016/j.ijnurstu.2004.06.011

[57] Marshall C, Rossman GB. Designing qualitative research (4th ed.). Sage. 2006.

[58] McLeroy KR, Bibeau D, Steckler A, Glanz K. Ecological perspective on promotion programs. Health Educ Q. 1988;15(4):351–77. 10.1177/109019818801500401. 3068205 10.1177/109019818801500401

[59] Millett GA, Jones AT, Benkeser D, Baral S, Mercer L, Beyrer C, Honermann B, Lankiewicz E, Mena L, Crowley JS, Sherwood J, Sullivan PS. Assessing differential impacts of COVID-19 on Black communities. Ann Epidemiol. 2020;47:37–44. 10.1016/j.annepidem.2020.05.003. 32419766 10.1016/j.annepidem.2020.05.003 PMC7224670

[60] Norberg AN, Norberg PRBM, Manhães FC, Filho RMF, de Souza DG, de Queiroz MMC, Neto CHG, Ribeiro PC, dos Boechat JCS, Viana KS, da Silva MTR. Public health strategies against social stigma in the Mpox Outbreak: A Systematic Review. J Adv Med Med Res. 2024;36(2):33–47. 10.9734/jammr/2024/v36i25365.

[61] Ogunbajo A, Euceda A, Smith J, Ekundayo R, Wattree J, Brooks M, Hickson D. Demographics and health beliefs of Black gay, bisexual, and other sexual minority men receiving a Mpox vaccination in the United States. J Urban Health. 2023. 10.1007/s11524-022-00712-9. 36662397 10.1007/s11524-022-00712-9 PMC9854406

[62] O’Neal J, da Rosa JL, Astin M, Folkes D, Crowder D, Holland DP. Reversing inequity in Mpox vaccine distribution, Fulton County, Georgia, June–September 2022. Am J Public Health. 2023;113(12):1263–6. 10.2105/AJPH.2023.307416. 37797279 10.2105/AJPH.2023.307416 PMC10632850

[63] Pacherie E. The phenomenology of action: a conceptual framework. Cognition. 2008;107(1):179–217. 10.1016/j.cognition.2007.09.003. 17950720 10.1016/j.cognition.2007.09.003

[64] Palamenghi L, Barello S, Boccia S, Graffigna G. Mistrust in biomedical research and vaccine hesitancy: the forefront challenge in the battle against COVID-19 in Italy. Eur J Epidemiol. 2020;35(8):785–8. 10.1007/s10654-020-00675-8. 32808095 10.1007/s10654-020-00675-8 PMC7431109

[65] Patton M. Qualitative research and evaluation methods (3rd ed.). Sage. 2002.

[66] Phillips G, Curtis MG, Felt D, Davoudpour S, Rodriguez-Ortiz AE, Cortez A, French AL, Hosek SG, Serrano PA. Changes in sexual behaviors due to Mpox: a cross-sectional study of sexual and gender minority individuals in Illinois. Prevention Science. 2023;25:628–37. 10.1007/s11121-023-01604-3. 37906357 10.1007/s11121-023-01604-3 PMC11112966

[67] Quinn SC, Andrasik MP. Addressing vaccine hesitancy in BIPOC communities — toward trustworthiness, partnership, and reciprocity. N Engl J Med. 2021;385(2):97–100. 10.1056/nejmp2103104. 33789007 10.1056/NEJMp2103104

[68] Rodriguez-Diaz CE, Crowley JS, Santiago-Rivera Y, Millett GA. From COVID-19 to monkeypox: unlearned lessons for Black, Latino, and other men with HIV who have sex with men. Am J Public Health. 2022;112(11):1564–620. 10.2105/AJPH.2022.307093. 36108251 10.2105/AJPH.2022.307093 PMC9558207

[69] Rose G. Situating knowledges: positionality, reflexivities and other tactics. Prog Hum Geogr. 1997;21(3):305–20. 10.1191/030913297673302122.

[70] Sah R, Reda A, Abdelaal A, Mohanty A, Siddiq A, Alshahrani NZ, Amer FA, Rodriguez-Morales AJ. A potential monkeypox pandemic: are we making the same mistakes as COVID-19? New Microbes and New Infections. 2022;49–50. 10.1016/j.nmni.2022.101030 10.1016/j.nmni.2022.101030 PMC9473140 36123971

[71] Sandelowski M. Sample size in qualitative research. Res Nurs Health. 1995;18(2):179–83. 10.1002/nur.4770180211. 7899572 10.1002/nur.4770180211

[72] Sandelowski M. Using qualitative research. Qual Health Res. 2004;14(10):1366–86. 10.1177/1049732304269672. 15538005 10.1177/1049732304269672

[73] Sandelowski M. Getting it right. Res Nurs Health. 2010;33(1):1–3. 10.1002/nur.20365. 20014032 10.1002/nur.20365

[74] Sandelowski M, Barroso J. Finding the findings in qualitative studies. J Nurs Scholarsh. 2002;34(3):213–9. 10.1111/j.1547-5069.2002.00213.x. 12237982 10.1111/j.1547-5069.2002.00213.x

[75] Siu A, Chan W. Unveiling racial and ethnic disparities in MPOX virus vaccine distribution and demographic patterns in the United States. Multidisciplinary Digital Publishing Institute. 2023. 10.20944/preprints202311.0275.v1

[76] Starks TJ, Scales D, Castiblanco J, Gorman J, Cain D. Correlates of Mpox vaccination among sexual minority men in the United States: sexual behavior, substance use, and main partner relationships. J Sex Res. 2023;60(5):634–44. 10.1080/00224499.2023.2188443. 36920105 10.1080/00224499.2023.2188443 PMC10175215

[77] Streubert-Speziale HJ, Carpenter DR. Qualitative research in nursing: advancing the humanistic imperative (3rd ed.). Lippincott Williams & Wilkins. 2003.

[78] Sultana F. Reflexivity, positionality and participatory ethics: negotiating fieldwork dilemmas in international research. ACME Int E-J Critical Geographics. 2007;6(3):374–85.

[79] Turan JM, Elafros MA, Logie CH, Banik S, Turan B, Crockett KB, Pescosolido B, Murray SM. Challenges and opportunities in examining and addressing intersectional stigma and health. BMC Med. 2019;17(1):1–15. 10.1186/s12916-018-1246-9. 30764816 10.1186/s12916-018-1246-9 PMC6376691

[80] Turpin RE, Mandell CJ, Camp AD, Davidson Mhonde RR, Dyer TV, Mayer KH, Liu H, Coates T, Boekeloo BO. Monkeypox-related stigma and vaccine challenges as a barrier to HIV pre-exposure prophylaxis among Black sexual minority men. Int J Environ Res Public Health. 2023;20(14). 10.3390/ijerph20146324 10.3390/ijerph20146324 PMC10378858 37510557

[81] Watson RJ, Chang CJ, Feinstein BA, Moody RL, Caba A, Eaton LA. PrEP stigma and logistical barriers remain significant challenges in curtailing HIV transmission among Black and Hispanic/Latinx cisgender sexual minority men and transgender women in the US. AIDS Care - Psychol Socio-Med Aspects of AIDS/HIV. 2022;34(11):1465–72. 10.1080/09540121.2022.2098908. 10.1080/09540121.2022.2098908 PMC9842805 35848490

[82] Webb Hooper M, Napoles A, Perez-Stable E. COVID-19 and racial/ethnic disparities. J Am Med Assoc. 2020;323(24):2466–7. 10.1002/jclp.20757. 10.1001/jama.2020.8598 PMC9310097 32391864

[83] Zelaya CE, Smith BP, Riser AP, Hong J, Distler S, Siobhán O’connor Belay E, Shoeb, Mohammad, Waltenburg MA, Negron ME, Ellington S. Urban and rural Mpox incidence among persons aged 15–64 years — United States, May 10–December 31, 2022. Morbidity and Mortality Weekly Report. 2023;72(21). https://wonder.cdc.gov/single-race-v2021.html 10.15585/mmwr.mm7221a2 PMC10231937 37227985

[84] Zhao T, Xuan K, Uy JP, Sun C. In the shadow of COVID-19: HIV-infected individuals need more attention. J Med Virology, 2020;26378. 10.1002/jmv.26378 10.1002/jmv.26378 32735365

